# Invasive *Fusarium* rhinosinusitis in COVID-19 patients: report of three cases with successful management

**DOI:** 10.3389/fcimb.2023.1247491

**Published:** 2023-09-14

**Authors:** Mahzad Erami, Shima Aboutalebian, Seyed Jamal Hashemi Hezaveh, Amir Hassan Matini, Mansooreh Momen-Heravi, Amir Hossein Ahsaniarani, Shirin Shafaee Arani, Mohsen Ganjizadeh, Hossein Mirhendi

**Affiliations:** ^1^ Infectious Diseases Research Center, Kashan University of Medical Sciences, Kashan, Iran; ^2^ Department of Medical Parasitology and Mycology, School of Medicine, Isfahan University of Medical Sciences, Isfahan, Iran; ^3^ Mycology Reference Laboratory, Research Core Facilities Laboratory, Isfahan University of Medical Sciences, Isfahan, Iran; ^4^ Department of Medical Parasitology and Mycology, School of Public Health, Tehran University of Medical Sciences, Tehran, Iran; ^5^ Department of Pathology and Histology, School of Medicine, Shahid Beheshti Hospital, Kashan University of Medical Sciences, Kashan, Iran; ^6^ Department of Infectious Disease, School of Medicine, Shahid Beheshti Hospital, Kashan University of Medical Sciences, Kashan, Iran; ^7^ Department of Otorhinolaryngology, School of Medicine, Matini Hospital, Kashan University of Medical Sciences, Kashan, Iran

**Keywords:** *Fusarium*, rhinosinusitis, COVID-19, immunocompromised, antifungal susceptibility testing

## Abstract

Invasive fungal rhinosinusitis (IFRS) is a life-threatening infection that can occur in immunocompromised patients, including those with COVID-19. Although Mucorales and *Aspergillus* species are the most common causes of IFRS, infections caused by other fungi such as *Fusarium* are rare. In this report, we present three cases of proven rhinosinusitis fusariosis that occurred during or after COVID-19 infection. The diagnosis was confirmed through microscopy, pathology, and culture, and species identification of the isolates was performed by DNA sequencing the entire ITS1-5.8 rRNA-ITS2 region and translation elongation factor 1-alpha (TEF-1α). Antifungal susceptibility testing was conducted according to CLSI guidelines. The causative agents were identified as *Fusarium proliferatum, F. oxysporum + Aspergillus flavus*, and *F. solani/falciforme.* Treatment involved the administration of antifungal medication and endoscopic sinus surgery to remove the affected mucosa, leading to the successful resolution of the infections. However, one patient experienced a recurrence of IFRS caused by *A. flavus* 15 months later. Early diagnosis and timely medical and surgical treatment are crucial in reducing mortality rates associated with invasive fusariosis. Additionally, the cautious use of corticosteroids in COVID-19 patients is highly recommended.

## Introduction

*Aspergillus* and Mucorales have traditionally been recognized as the main culprits behind invasive mold infections. However, there has been a concerning increase in the occurrence of fungal infections caused by other pathogens including *Fusarium*, which have shown alarmingly high mortality rates. *Fusarium* species can be found widely in nature, including in soil, decaying plants (Nucci and Anaissie, 2007), and organic substrates and may enter the human body through various routes, including the airways, skin breakdown, and mucosal membranes (Carneiro et al., 2011). They are opportunistic pathogens capable of causing a range of infections in humans, with the outcome largely dependent on the immune status of the host and the site of infection (Nucci and Anaissie, 2007; Campo et al., 2010). *Fusarium* infections can be classified into three main groups. Superficial infections such as keratitis and onychomycosis, localized infections such as allergic or invasive sinusitis, and disseminated infections which are the most severe clinical form and often lead to mortality rates ranging from 60% to 100%. The disseminated infections primarily occur in severely immunocompromised patients, including those with hematological malignancies or undergoing hematopoietic stem cell transplantation (Nucci and Anaissie, 2007). Additionally, *Fusarium* infections can manifest in less common forms such as otitis, osteomyelitis, sinusitis, arthritis, and brain abscesses (Nucci and Anaissie, 2007; Thomas et al., 2020).

The genus *Fusarium* belongs to the order Hypocreales and encompasses over 300 species, categorized into more than 20 complexes of related species (O’Donnell et al., 2013; O’Donnell et al., 2020). The species that predominantly cause diseases in humans include *F. solani, F. oxysporum*, and *Gibberella fujikuroi/F. fujikuroi* species complexes (Al-Hatmi et al., 2018). Among these, *F. solani* stands out as the most common and virulent species, accounting for approximately 40-60% of all infections (Mayayo et al., 1999).

The emergence of COVID-19 has posed a significant threat to individuals with compromised immune systems, including those with conditions such as diabetes mellitus, leukemia, organ transplant, AIDS, and individuals undergoing high-dose corticosteroid treatment. In the context of COVID-19, invasive fungal infections have become a notable concern, primarily attributed to *Aspergillus*, Mucorales, and *Candida* species (Hoenigl et al., 2022). Fungal rhinosinusitis can arise from various fungal species, each requiring specific treatment approaches. It is crucial to promptly diagnose and accurately identify these pathogenic fungi for effective clinical decision-making and to study the clinical and microbial epidemiology of fungal infections (Vennewald et al., 1999).

In our previous cross-sectional study conducted at Shahid-Beheshti Hospital in Kashan, Iran, between January 2020 and July 2022, we examined the occurrence of COVID-19-associated invasive fungal rhinosinusitis (IFRS) and investigated three cases of *Fusarium* rhinosinusitis (Erami et al., 2023a). The present report describes the details of these three cases that are documented instances of COVID-19-associated rhinosinusitis caused by *Fusarium* species, and underscores the emergence of *Fusarium* as a potential pathogen in immunocompromised individuals and those affected by COVID-19.

## The cases report

### Case 1

**Figure 1:**
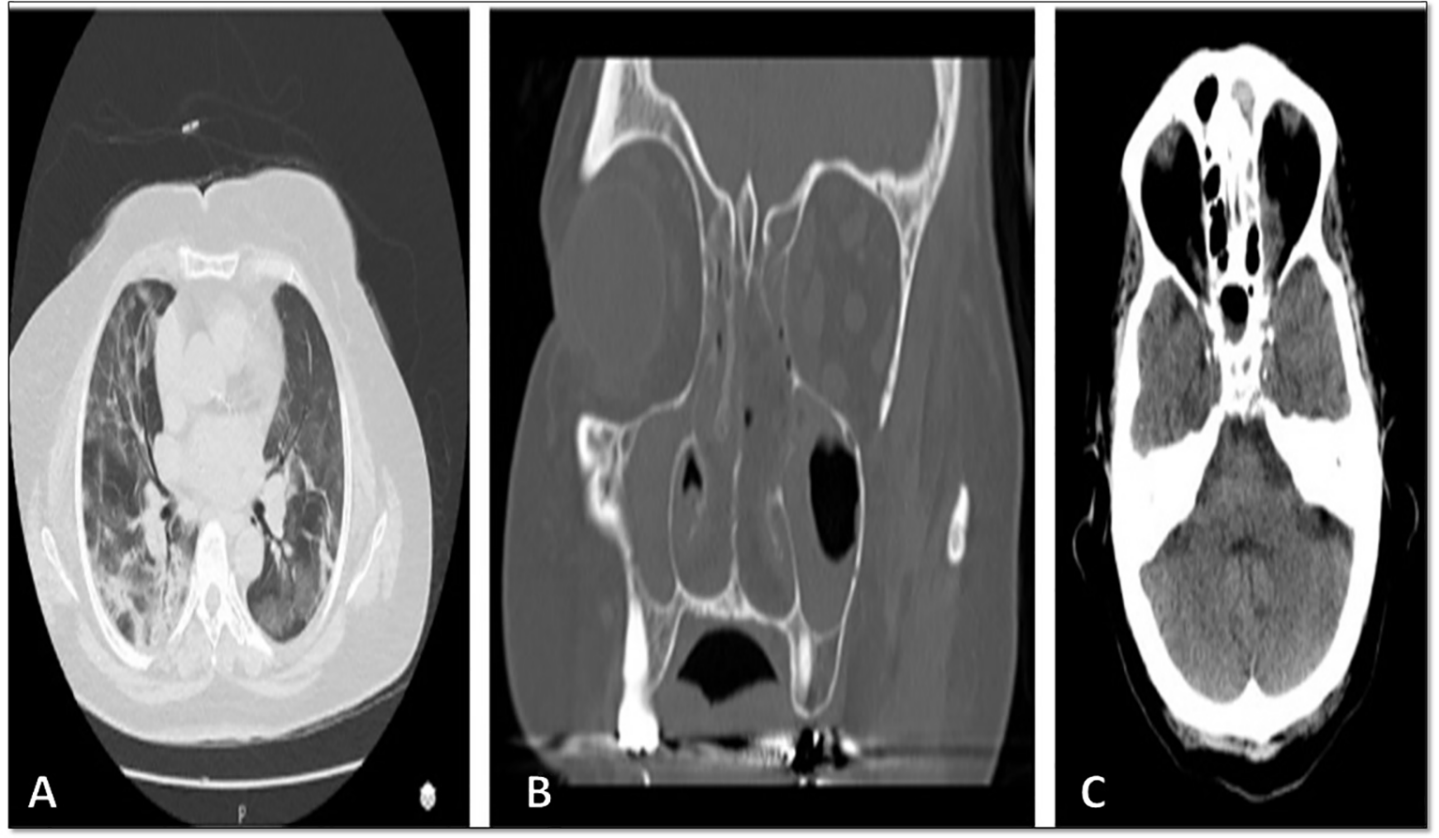
**(A)** Chest CT scan showing patchy ground-glass opacity, bilateral consolidation, and atelectatic bands. **(B, C)** Paranasal and orbital CT scan displaying opacification in all paranasal sinuses, destruction of medial septum in the left maxilla, bilateral retrolental fat haziness, medial extra and intraconal fat haziness, and inflammation of medial and superior rectus muscles. These findings suggest a fungal sinonasal invasion in the right orbital.

On September 5, 2021, a 68-year-old female who had previously recovered from COVID-19 pneumonia 40 days prior and had a history of receiving remdesivir (200 mg on the first day and then 100 mg for 5 days) and dexamethasone (8 mg intravenously daily for 5 days), was admitted to Shahid-Beheshti Hospital in Kashan, Iran. She presented with swelling on the right side of her face, redness around the eyes, right upper eyelid edema, right total ophthalmoplegia, and no perception of light. The patient had a medical history of hypertension, hyperlipidemia, anemia, pancytopenia, and heart failure. During the physical examination, her oxygen saturation (SpO2) level was 70%, respiratory rate was 19 breaths per minute, blood pressure was 92/56 mmHg, and body temperature was 36°C. Initial laboratory results showed fasting blood sugar of 84 mg/dL (70-115 mg/dL), sodium of 136 mmol/L (135-145 mmol/L), potassium of 3.3 mmol/L (3.5-5.3 mmol/L), calcium of 8.2 mg/dL (8.6-10.6 mg/dL), creatinine of 1 mg/dL (0.4-1.5 mg/dL), and elevated levels of lactic dehydrogenase (LDH) at 910 IU (<550 IU). The patient had a low white blood cell count of 3.6 × 10^3^/µL (4–11 × 10^3^/µL), neutrophil count of 79% (35-80%), lymphocyte count of 14% (18-44%), hemoglobin of 6 g/dL (11.7–15.5 g/dL), platelet count of 108 × 10^3^/µL (165-415 × 10^3^/µL), D-dimer level of 867 ng/mL (<500), erythrocyte sedimentation rate (ESR) of 80 mm in the first hour, and C-reactive protein (CRP) of 102 mg/L (negative: <8). Blood and urine cultures were negative for bacteria and fungi. Despite the increase in inflammatory biomarkers, the patient tested negative for COVID-19 via RT-PCR and was suspected to have bacterial superinfection. As a result, broad-spectrum antibacterial therapy including vancomycin, ciprofloxacin, and imipenem was initiated. A chest CT scan revealed a mix of patchy ground-glass opacity (GGO), bilateral consolidation, and atelectatic bands (**Figure 1A**).

The CT scan of the paranasal sinuses and orbits revealed opacification in all paranasal sinuses, destruction of the medial septum of the left maxilla, and bilateral haziness of the retrobulbar and intraconal fat with involvement of the medial extraocular muscles. These findings indicated a fungal invasion of the sinonasal region in the right orbit (**Figures 1B, C**). The patient was initiated on empirical treatment with liposomal amphotericin B (300 mg/day) and underwent endoscopic surgical debridement.

**Figure 2:**
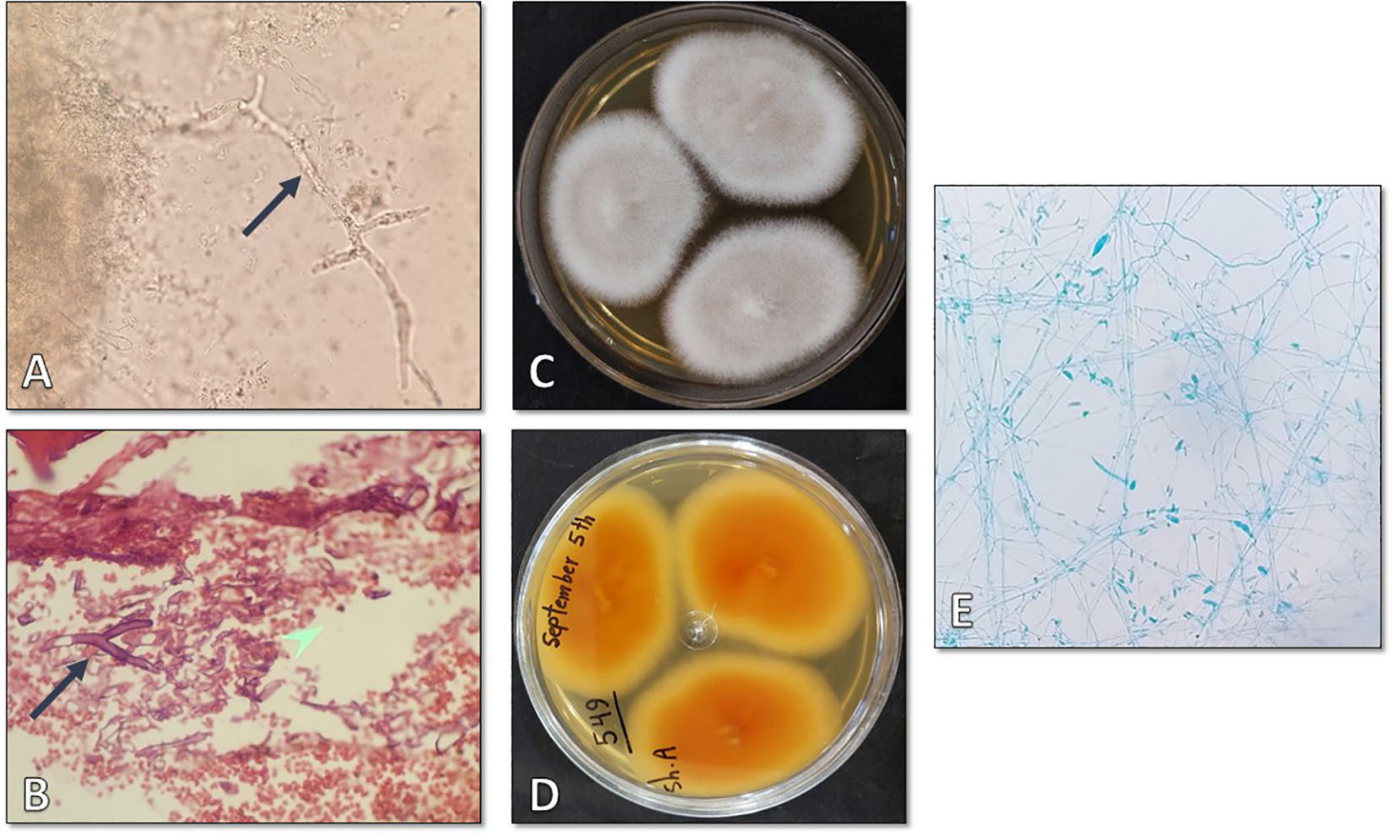
**(A)** Direct microscopic examination using 20% KOH revealing hyaline hyphae (×400). **(B)** Histopathologic examination with H&E staining showing infiltration of acute and chronic inflammatory cells, bleeding, and fungal hyphae (×1000). **(C, D)** Surface and reverse isolated colony after four days of incubation at 25°C. **(E)** Microscopic appearance of the isolate as observed on a slide prepared from the fungal culture (×400).

Microscopic examination of the clinical specimens obtained from the paranasal sinuses and necrotic tissues using KOH preparation showed the presence of hyaline hyphae (**Figure 2A**). Histopathological examination with hematoxylin and eosin (H&E) staining revealed infiltration of acute and chronic inflammatory cells, bleeding, and presence of fungal hyphae (**Figure 2B**). Three inoculums were cultured on Sabouraud dextrose agar (SDA) plates, resulting in colonies with abundant aerial mycelium that appeared white and cottony (**Figures 2C, D**). Slide culture and aniline blue staining of the colonies indicated the presence of *Fusarium* spp. (**Figure 2E**). The patient underwent postoperative treatment with amphotericin B for a duration of two weeks. Subsequently, she received posaconazole at a dosage of 300 mg per day for three weeks. After effectively managing her medical conditions, she was discharged and commenced a five-week regimen of oral voriconazole. Close clinical and radiological monitoring were implemented during this period. Over the course of sixteen months of follow-up, the patient’s initial symptoms resolved, and her overall condition improved significantly.

### Case 2

**Figure 3:**
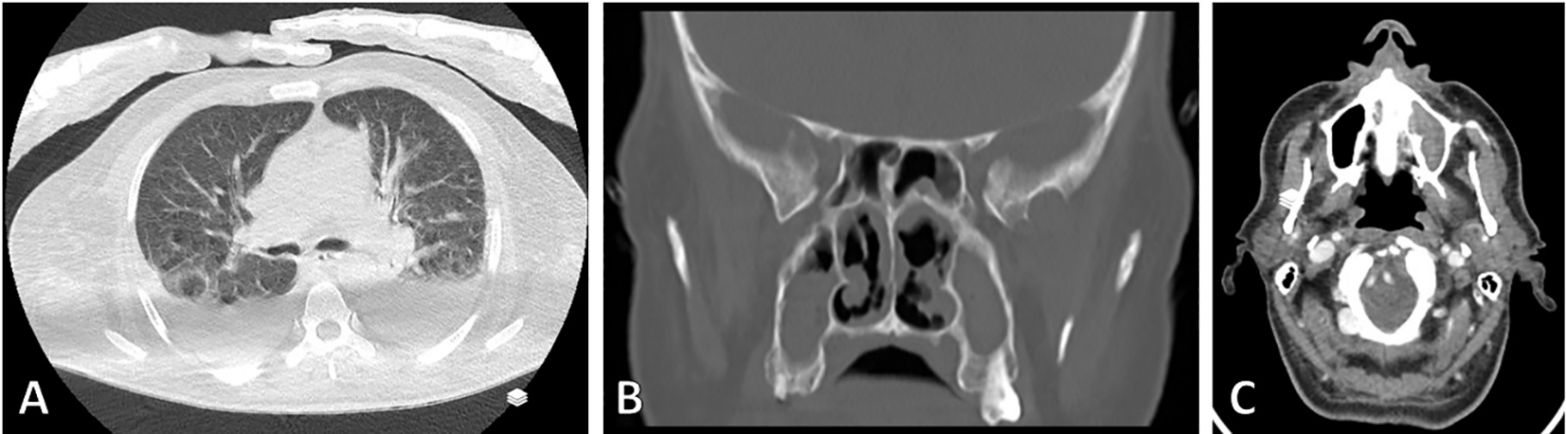
**(A)** Chest computed tomography scan demonstrating atelectasis in the right lower lobe, patchy consolidation in the left upper lobe, bilateral patchy ground-glass opacity, bilateral pleural effusion, pericardial effusion, right collapse consolidation, and air bronchogram. **(B, C)** Paranasal CT scan showing bilateral medial and sphenoid opacification, ethmoid destruction, medial septum destruction in the left maxilla, and bilateral frontal mucosal thickening.

On July 11, 2021, a 61-year-old farmer with a medical history of acute myeloid leukemia (AML), diabetes mellitus, hypertension, and invasive aspergillosis of the paranasal sinuses, was admitted to Shahid-Beheshti Hospital in Kashan, Iran. The reason for admission was progressive dyspnea caused by confirmed COVID-19. During the physical examination, the patient’s body temperature was measured at 36.8°C, blood pressure at 130/80 mm Hg, respiratory rate at 16 breaths per minute, pulse rate at 80 beats per minute, and oxygen saturation (SpO2) at 96% while breathing ambient air. A chest computed tomography (CT) scan conducted on day 1 revealed multiple abnormalities, including atelectasis in the right lower lobe (RLL), patchy consolidation in the left upper lobe (LUL), patchy ground-glass opacities (GGO) bilaterally, bilateral pleural effusion, pericardial effusion, right collapse consolidation, and air bronchogram (**Figure 3A**). The patient’s COVID-19 infection was confirmed through RT-PCR assay of nasopharyngeal and oropharyngeal swabs. Laboratory findings indicated a total white blood cell count of 14.8, with lymphocytes comprising 11% (18-44%) and neutrophils comprising 88% (35-80%). The platelet count was 205 × 10^3^/µL (165-415 × 10^3^/µL), hemoglobin level was 10.7 g/dL (11.7–15.5 g/dL), fasting blood sugar was 337 mg/dL (70-115 mg/dL), hemoglobin A1C was 8.7% (for non-diabetic individuals: 4.4-6.7%), and serum creatinine level was 0.8 mg/dL (0.4-1.5 mg/dL). Additionally, the patient had an elevated serum ferritin level of 401 ng/mL, an erythrocyte sedimentation rate (ESR) of 71 mm in the first hour, C-reactive protein (CRP) level of 177 mg/L (<8), lactate dehydrogenase (LDH) level of 10234 IU (<550 IU), and D-dimer level of 1087 ng/mL (negative: <500). While the enzyme-linked immunosorbent assay (ELISA) for Galactomannan in the serum was negative, the blood culture yielded positive results for *Enterococcus* species.

On the first day of hospital admission, the patient was prescribed metformin (500 mg/day) for diabetes mellitus and received standard combination chemotherapy for AML. In addition, he underwent empirical antibiotic treatment with meropenem and vancomycin, antiviral therapy with remdesivir (100 mg), and received dexamethasone (8 mg intravenously/twice daily). However, by the third day of hospitalization, his condition deteriorated, leading to progressive hypoxemia. As a result, he was transferred to the intensive care unit (ICU) and required invasive mechanical ventilation. Given the worsening lung involvement caused by COVID-19 infection, the patient was administered a single dose of tocilizumab (125 mg/day). Despite the treatment, there were no signs of recovery observed during the second and third weeks of hospitalization. On the twenty-fourth day after developing COVID-19, the patient began experiencing various symptoms including headache, weakness, retro-orbital pain, a bruised face, and facial numbness. A paranasal CT scan revealed opacification in bilateral medial, sphenoid, and ethmoid sinuses, as well as destruction of the medial septum in the left maxilla and nasal septum, and bilateral frontal mucosal thickening (**Figures 3B, C**). However, no rhino-orbital or cerebral abnormalities were identified. Despite receiving empiric treatment with liposomal amphotericin B (250 mg), the patient’s medical condition continued to significantly deteriorate.

**Figure 4:**
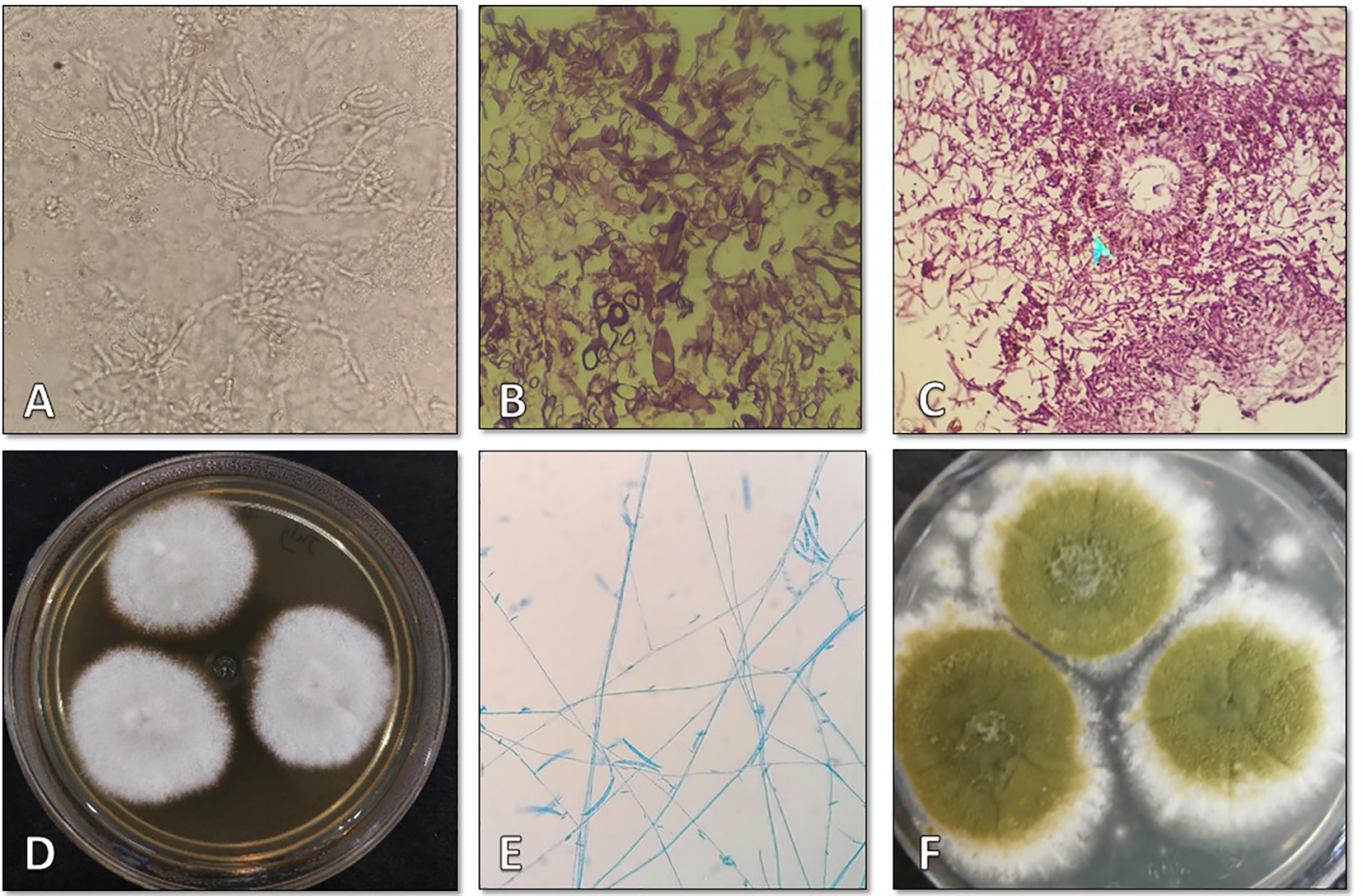
**(A)** Direct microscopic examination using 20% potassium hydroxide revealed septated hyaline hyphae (×400). **(B, C)** Histopathologic examination with hematoxylin-eosin stain showing infiltration of inflammatory cells (PMNs), fibrinolocyte exudate, septate acute angle fungal hyphae, and conidial head (×1000). **(D, F)** Morphological characteristics of colonies on Sabouraud dextrose agar after four days of incubation at 25 °C. **(E)** Microscopic morphology of *Fusarium* observed at ×400 magnification.

On day 34, a spiral CT scan of the brain revealed senile calcification in the basal ganglia and hyperdense opacification, including air bubbles in the maxillary and sphenoid sinuses. On day 35, the patient underwent endoscopic sinus and nasal surgery. During the procedure, necrotic tissue was removed, and KOH microscopy examination (**Figure 4A**) identified the presence of hyaline septate hyphae. Histopathology examination showed infiltration of inflammatory cells (PMNs), fibrinolocyte exudate, septate acute-angle fungal hyphae, and conidial head (**Figures 4B, C**). The isolated colony grown on SDA culture exhibited abundant white cottony mycelium and a purple undersurface (**Figure 4D**), and microscopic examination of the colonies confirmed the presence of *Fusarium* spp. (**Figure 4E**). Additionally, colonies of *Aspergillus flavus* with a yellow-green surface, white border, and velvety to cottony texture were observed (**Figure 4F**).

Following debridement, the patient was prescribed oral voriconazole (200 mg/12 h), and his condition gradually improved. He was eventually discharged from the ICU and the hospital and started a 6-week course of oral voriconazole with close clinical and radiological monitoring. Surprisingly, after 15 months, he experienced symptoms of IFRS again. Endoscopy and debridement were performed, and KOH examination and histopathology revealed the presence of hyaline septate hyphae. Over the seven-month follow-up period, the patient reported no recurrence of symptoms and confirmed complete recovery.

### Case 3

**Figure 5:**
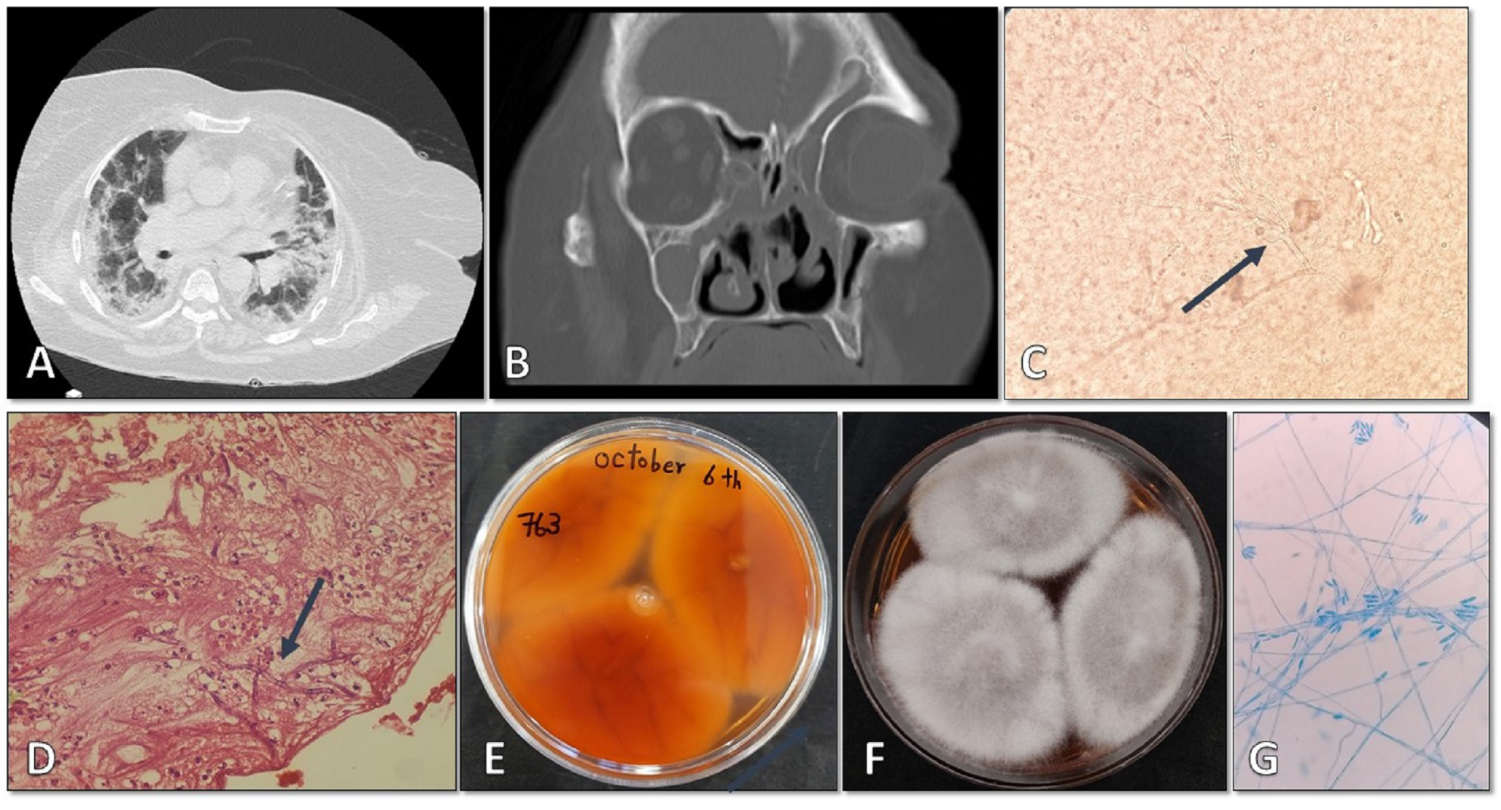
**(A)** Chest computed tomography scan showing bilateral disseminated mixed ground glass and consolidation suggestive of COVID-19. **(B)** Paranasal sinuses demonstrating fibrino leukocytic exudate, necrosis, and destruction of the nasal septum. **(C)** Direct KOH microscopy of tissue samples revealing hyaline septate hyphae (×400). **(D)** Histopathological examination of tissue sections revealing necrosis, edema, bleeding, and irregular and acute angle septate hyphae (×1000). **(E, F)** Reverse and surface of the isolated *Fusarium solani* colony on Sabouraud dextrose agar after four days of incubation at 25°C. **(G)** Microscopic morphological characteristics of the isolate in slide culture at ×400 magnification.

On October 6, 2021, a 69-year-old woman with multiple comorbidities including diabetes mellitus, hypertension, anemia, heart failure, chronic kidney disease, and hyperlipidemia, was referred to Shahid-Beheshti Hospital in Kashan, due to mild respiratory symptoms persisting for one month. Her symptoms included fever, weakness, myalgia, and cough. Upon admission, her vital signs were as follows: body temperature of 37.8°C, blood pressure of 150/77 mmHg, pulse rate of 18 beats per minute, respiratory rate of 18 breaths per minute, and SpO2 of 78-80% on ambient air. Laboratory tests revealed abnormal findings, including an elevated fasting blood sugar level of 296 mg/dL (70-115 mg/dL), hemoglobin A1C level of 7.1% (non-diabetic: 4.4-6.7%), triglyceride level of 217 mg/dL (<200), creatinine level of 1.7 mg/dL (0.4-1.5 mg/dL), blood urea nitrogen level of 50 mg/dL (7-23 mg/dL), LDH level of 603 IU (<550 IU), CRP level of 146 mg/L (<8), ESR level of 60 mm in the first hour, white cell count of 5.6 × 10^3/μL (4–11 × 10^3/μL), lymphocyte count of 10% (18-44%), neutrophil count of 85% (35-80%), red cell count of 3.55 × 10^6/μL (3.9-5.8 × 10^6/μL), platelet count of 138 × 10^3^/µL (165-415 × 10^3^/µL), and hemoglobin level of 7.6 g/dL (11.7–15.5 g/dL). The laboratory blood analysis also revealed lymphopenia and a significant increase in CRP. Urine and blood cultures for bacteria and fungi, as well as ELISA for Galactomannans, were negative. However, the PCR assay on naso/oropharyngeal swabs was positive for COVID-19. A chest CT scan revealed bilateral disseminated mixed ground glass opacities and consolidations, indicating a possible case of COVID-19 (**Figure 5A**). Subsequently, the patient’s treatment regimen was initiated, consisting of remdesivir at an initial dose of 200 mg followed by 100 mg for 10 days, along with dexamethasone administered at a daily dosage of 8 mg. Paranasal endoscopy revealed the presence of fibrino leukocytic exudate and necrosis, along with the destruction of the nasal septum (**Figure 5B**). Surgery was scheduled to remove the necrotic tissue. KOH direct microscopy of the surgical biopsy specimens revealed the presence of hyaline septate hyphae (**Figure 5C**), and histopathology showed evidence of necrosis, edema, bleeding, as well as irregular and acute-angle septate hyphae (**Figure 5D**). The biopsy sample cultured on SDA agar plates initially produced white and cottony colonies that rapidly developed a violet center with a lighter periphery (**Figure 5E**), with the reverse side appearing light but deeply colored (**Figure 5F**). Slide culture and aniline blue staining of the colonies confirmed the microscopic morphology indicative of *Fusarium* species (**Figure 5G**).

The patient initially received intravenous (IV) liposomal amphotericin B; however, there was no observed improvement in symptoms, and radiography did not indicate any signs of improvement. Due to the disease progression and worsening hypoxia, dexamethasone was switched to methylprednisolone at a daily dose of 125 mg. Additionally, the patient commenced treatment with intravenous tocilizumab at a dosage of 400 mg.

The patient underwent multiple surgeries to remove the necrotic sinus tissue, but during the last debridement, she experienced severe bleeding, resulting in severe hypoxia, decreased level of consciousness, mechanical ventilation, and transfer to the ICU. After several days of treatment with amphotericin B, *Fusarium* species were identified in the tissue culture. Consequently, the treatment was switched to IV voriconazole at a dose of 6 mg/kg every 12 hours. After five weeks of voriconazole treatment, the patient’s symptoms improved, and subsequent follow-up radiographs showed a decrease in the extent of the lesions. Finally, after two months, the patient was discharged from the hospital and recommended to have regular follow-ups with an endocrinologist and ENT specialist. After a period of 15 months, the patient remained asymptomatic for the infection.

### Species identification of the isolates

**Figure 6:**
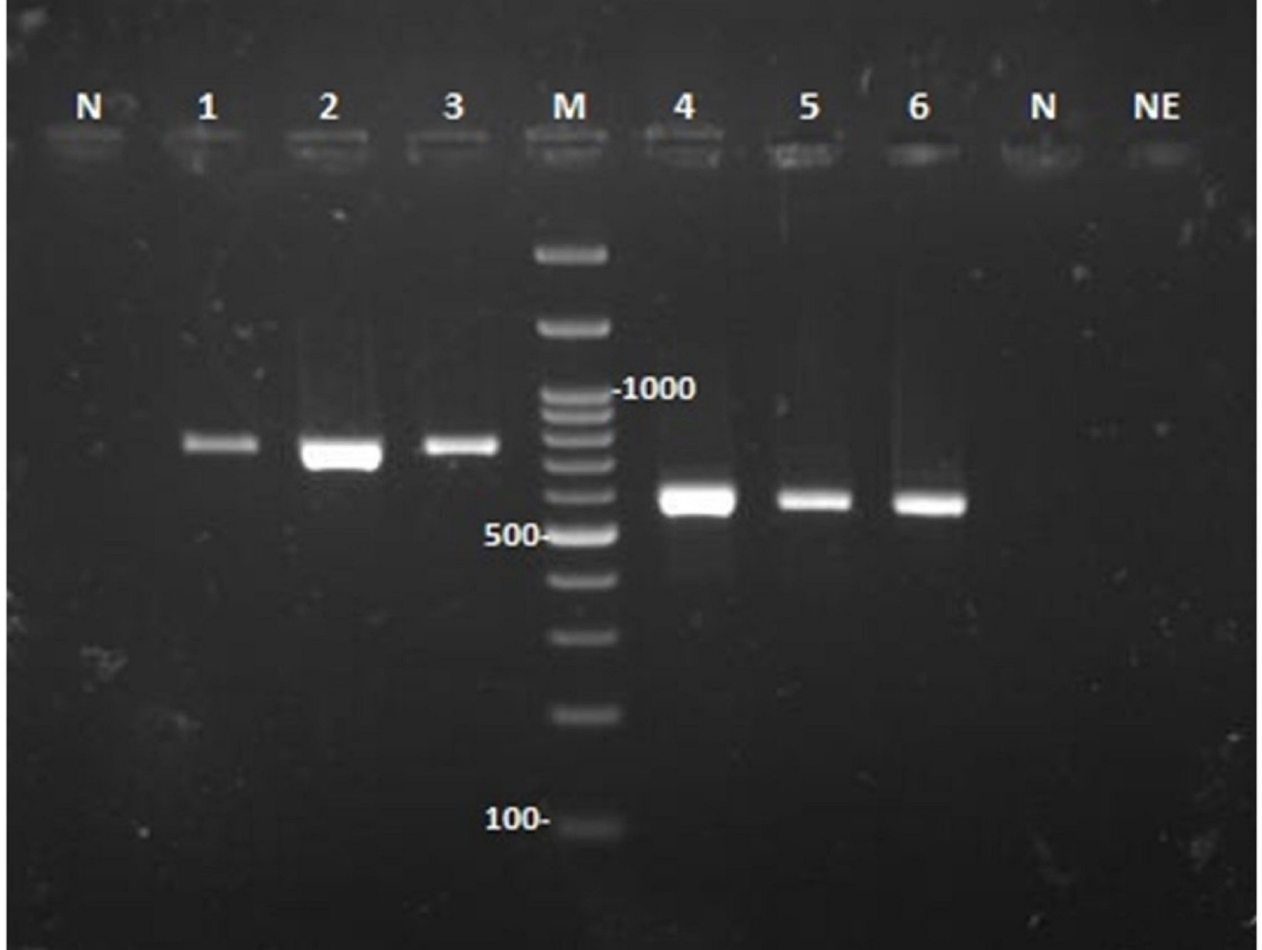
Agarose gel electrophoresis of PCR products from DNA amplification of three *Fusarium* strains isolated from three patients. Amplification was performed for the translation elongation factor 1-alpha (lanes 1-3) and the entire ITS1-5.8 rRNA-ITS2 region (lanes 4-6). Lanes N and NE denote the PCR and DNA extraction controls, respectively. Lane M displays a 100 bp DNA molecular size marker.

Genomic DNAs from the colonies isolated from the patients were extracted using glass-bead preparation followed by phenol-chloroform purification as previously described (Aboutalebian et al., 2021). The primers ITS1 (TCCGTAGGTGAACCTGCGG) and ITS4 (TCCTCCGCTTATTGATATGC) (Aboutalebian et al., 2022) were used to amplify the entire internal transcribed spacer (ITS) region of the nuclear ribosomal DNA for all isolates, and the primers EF1 (ATGGGTAAGRAGACAAGAC) and EF2 (GGARGTACCAGTSATCATGTT) (O'Donnell et al., 1998) were used to amplify translation elongation factor 1-alpha (TEF-1α) for *Fusarium* isolates (**Figure 6**). The resulting PCR products, approximately 600 bp and 700-800 bp in size, were purified and sequenced at the Core Facilities Research Laboratory, Isfahan, Iran, using BigDye Terminators (Applied Biosystems). The sequences were then analyzed for their identity using the BLAST program (https://blast.ncbi.nlm.nih.gov/Blast.cgi). The causative agents for cases 1, 2, and 3 were identified as *Fusarium proliferatum*, a combination of *Fusarium oxysporum* and *Aspergillus flavus*, and *Fusarium solani/falciforme*, respectively.

### Antifungal susceptibility testing

AFST was conducted on the isolated *Fusarium* colonies. To maximize conidial harvest, the colonies were subcultured on potato dextrose agar (PDA) and incubated at 35°C for up to 48-72 hours and a suitable inoculum (0.4 - 5 × 10^4 CFU/ml) were prepared. The AFST followed the broth microdilution method based on CLSI, M38-3rd, for amphotericin B, itraconazole, voriconazole, and posaconazole. Minimum inhibitory concentration (MIC) results were visually determined after 24 hours of incubation and are presented in **Table 1**. Quality controls were included in the tests, using *Candida parapsilosis* ATCC 22019 and *Candida krusei* ATCC 6258.

**Table 1:** Characteristics of three COVID-19 patients co-infected with invasive fungal rhinosinusitis (IFRS) caused by *Fusarium*.

	Case 1	Case 2	Case 3
**Sex**	Female	Male	Female
**Age (year)**	68	61	69
**Underlying condition**	Hypertension Heart failure Anemia Hyperlipidemia	Malignancy Diabetes mellitus Hypertension	Diabetes mellitus Hypertension Heart failure Chronic kidney disease Hyperlipidemia Anemia
**Site (s) of infection**	Rhino-sinusal + orbital	Rhino-sinusal	Rhino-sinusal
**Treatment with steroids**	Yes	Yes	Yes
**Clinical manifestations**	Headache Ptosis and eyelid Swelling Retro-orbital pain Diminution of vision Nasal regurgitation	Headache Retro-orbital pain Facial numbness	Headache Ptosis and eyelid swelling Retro-orbital pain Facial numbness
**Duration of illness (day)**	37	64	64
**Debridement**	Yes	Yes	Yes
**Mechanical ventilation**	No	Yes	No
**Antifungal medication**	Liposomal amphotericin B Posaconazole Voriconazole	Liposomal amphotericin B Voriconazole	Liposomal amphotericin B Voriconazole
**Outcome**	Cured	Cured	Cured
**Causative agent (**Target: Internal transcribed spacer)	*Fusarium proliferatum*	*Fusarium oxysporum* + *Aspergillus flavus*	*Fusarium solani*/*falciforme*
**Causative agent (**Target: Translation elongation factor**)**	*Fusarium proliferatum*	*Fusarium oxysporum*	*Fusarium solani*/*falciforme*
**MIC for Amphotericin B, Itraconazole, Voriconazole, and Posaconazole (μg/mL) for *Fusarium* species, respectively**	4, >16, 8, and >16	8, >16, 4, and 8	4, >16, 4, and >16

**Table 1** provides a summary overview of the demographic, clinical, and diagnostic characteristics of the patients, as well as the results of the AFST conducted on the *Fusarium* isolates.

## Discussion

The incidence, morbidity, and mortality of IFRS have shown a significant increase in recent years. This can be attributed partly to the rising prevalence of immunodeficiency, the emergence of COVID-19, and advancements in the diagnosis of pathogenic fungi. While *Aspergillus* and Mucorales are the primary agents of IFRS, other fungi such as *Fusarium*, *Lomentospora, Curvularia, Alternaria, Cladosporium, Bipolaris, Exherosilum, Trichoderma, Schizophyllum, Candida*, and *Trichosporon* are occasionally reported as well (Ardeshirpour et al., 2014; Vinh et al., 2017; Shamsaei et al., 2021; Erami et al., 2023a; Erami et al., 2023b; Salah et al., 2023). However, the limited availability of diagnostic tools often results in delays in accurate detection and identification of invasive fungal pathogens, leading to delayed treatment. IFRS shares clinical features and predisposing risk factors, and the causal agents like *Aspergillus*, *Fusarium*, and other septate molds cannot be differentiated through direct microscopy and histopathology examination of the clinical samples alone.

In this study, we present three cases of rhinosinusitis infection caused by *Fusarium* species in COVID-19 patients. We identified the *Fusarium* colonies isolated from the patients using both ITS-sequencing and TEF1-α-sequencing. TEF1-α gene is currently the genetic marker that provides the highest resolution for species identification of *Fusarium* species (Raja et al., 2017).

AFST studies have shown significant variability in the minimum inhibitory concentration (MIC) range of *Fusarium* species, which is partly dependent on the specific species involved (Oechsler et al., 2013) *Fusarium* species are inherently resistant to echinocandins (Al-Hatmi et al., 2016), and some isolates also exhibit resistance to azoles (Batista et al., 2020). Given that liposomal amphotericin B and voriconazole are the preferred drugs for treating deep and disseminated *Fusarium* infections (Hoenigl et al., 2021), these drugs were empirically prescribed for our cases. Otolaryngologists performed endoscopic sinus surgery with debridement, removing mucosa, necrotic tissue, and healthy surrounding tissue to prevent further spread to adjacent tissues. Fortunately, the management was successful, and there were no fatalities. However, due to the intrinsic resistance of most strains and the absence of susceptibility breakpoints defined by the Clinical and Laboratory Standards Institute CLSI (Espinel-Ingroff et al., 2016), we were unable to determine susceptibility or resistance.

In all three cases, inflammatory markers were significantly elevated, while lymphocyte counts were decreased. The presence of COVID-19 may explain these findings, as the virus can cause lymphocyte destruction during the acute phase, thereby predisposing patients to co-infections. Furthermore, COVID-19 itself can act as an independent risk factor for developing fungal infections through mechanisms such as impaired immune response, prolonged corticosteroid therapy, virus-induced alveolar damage, and lymphopenia (Aboutalebian et al., 2023; Cattaneo et al., 2023). Apart from COVID-19, other notable risk factors in these cases included diabetes, corticosteroid use, and hypertension. CT scans revealed mucosal thickening, erosion of the hard palate, and mucosal necrosis in all cases. Case 2 had acute myeloid leukemia (AML), while cases 1 and 3 had severe anemia.

Only a limited number of documented cases of IFRS caused by *Fusarium* exist. In this study, we present three cases of *Fusarium* rhinosinusitis as secondary infections during or following COVID-19, which were attributed to *F. proliferatum, F. oxysporum*, and *F. solani/falciforme*. To the best of our knowledge, there is only one previously reported case of IFRS caused by *F. solani*. Dallé Rosa et al. (2018) described a case of IFRS resulting from *F. riograndense* (a newly identified species within the *F. solani* complex) infection in an 11-year-old boy with acute lymphoblastic leukemia, who was treated with voriconazole (Dallé Rosa et al., 2018). Additionally, there are only two reported cases of IFRS caused by *F. oxysporum*, and no clinical details are available for these cases (Shamsaei et al., 2021). Furthermore, there have been just four cases, including our current case 1, of IFRS caused by *F. proliferatum* with a presentation of rhinosinusitis. These cases involved two female patients from Iran and two from France, ranging in age from 21 to 68 years, with a mean age of 49.8 years. One patient experienced anosmia (Hashemi et al., 2015), another had esophagitis (Radulesco et al., 2019), while one of them had comorbidities such as coronary artery disease, dyslipidemia, and obesity (Radulesco et al., 2019). Our case had diabetes mellitus, hypertension, heart failure, and anemia. In all instances, surgical intervention was necessary to remove affected mucosa and necrotic tissue, resulting in a complete resolution of symptoms.

## Conclusion

In this study, three cases of rhinosinusitis infection caused by *Fusarium* species in COVID-19 patients are described. The high mortality rate associated with invasive fusariosis highlights the importance of early diagnosis, prompt medical and surgical intervention, and careful use of corticosteroids in COVID-19 patients to reduce mortality.

## Data availability statement

The original contributions presented in the study are included in the article/supplementary materials. Further inquiries can be directed to the corresponding author.

## Ethics statement

The studies involving humans were approved by the ethics committee of Tehran University of Medical Sciences, Tehran, Iran (IR.TUMS.SPH.REC.1399.329). The studies were conducted in accordance with the local legislation and institutional requirements. The participants provided their written informed consent to participate in this study. Written informed consent was obtained from the individual(s), and minor(s)’ legal guardian/next of kin, for the publication of any potentially identifiable images or data included in this article.

## Author contributions

SA and ME conducted the experiments and participated in data collection. SA drafted the manuscript, analyzed and interpreted the data. SH, AM, MM-H, AA, SSA, and MG contributed to collecting the clinical isolate and data collection. HM supervised all aspects of the study and critically reviewed the manuscript. All authors thoroughly reviewed and approved the final version of the manuscript.
